# Olive Tree Biovolume from UAV Multi-Resolution Image Segmentation with Mask R-CNN

**DOI:** 10.3390/s21051617

**Published:** 2021-02-25

**Authors:** Anastasiia Safonova, Emilio Guirado, Yuriy Maglinets, Domingo Alcaraz-Segura, Siham Tabik

**Affiliations:** 1 Laboratory of Deep Learning, Siberian Federal University, 660074 Krasnoyarsk, Russia; 2 Institute of Space and Information Technologies, Siberian Federal University, 660074 Krasnoyarsk, Russia; ymaglinets@sfu-kras.ru; 3 Department of Computer Science and Artificial Intelligence, University of Granada, 18071 Granada, Spain; siham@ugr.es; 4 Multidisciplinary Institute for Environment Studies “Ramón Margalef”, University of Alicante, 03690 Alicante, Spain; e.guirado@ual.es; 5 Department of Botany, Faculty of Science, University of Granada, 18071 Granada, Spain; dalcaraz@ugr.es; 6 iEcolab, Inter-University Institute for Earth System Research, University of Granada, 18006 Granada, Spain

**Keywords:** instance segmentation, machine learning, deep neural networks, olive trees, ultra-high resolution images

## Abstract

Olive tree growing is an important economic activity in many countries, mostly in the Mediterranean Basin, Argentina, Chile, Australia, and California. Although recent intensification techniques organize olive groves in hedgerows, most olive groves are rainfed and the trees are scattered (as in Spain and Italy, which account for 50% of the world’s olive oil production). Accurate measurement of trees biovolume is a first step to monitor their performance in olive production and health. In this work, we use one of the most accurate deep learning instance segmentation methods (Mask R-CNN) and unmanned aerial vehicles (UAV) images for olive tree crown and shadow segmentation (OTCS) to further estimate the biovolume of individual trees. We evaluated our approach on images with different spectral bands (red, green, blue, and near infrared) and vegetation indices (normalized difference vegetation index—NDVI—and green normalized difference vegetation index—GNDVI). The performance of red-green-blue (RGB) images were assessed at two spatial resolutions 3 cm/pixel and 13 cm/pixel, while NDVI and GNDV images were only at 13 cm/pixel. All trained Mask R-CNN-based models showed high performance in the tree crown segmentation, particularly when using the fusion of all dataset in GNDVI and NDVI (F1-measure from 95% to 98%). The comparison in a subset of trees of our estimated biovolume with ground truth measurements showed an average accuracy of 82%. Our results support the use of NDVI and GNDVI spectral indices for the accurate estimation of the biovolume of scattered trees, such as olive trees, in UAV images.

## 1. Introduction

Most of the world’s olive oil—around 2 million tones (66% of global production)—is produced in the European Union. Although recent intensification techniques organise olive trees in hedgerows [1], most olive groves are rainfed and trees are planted at ~6 m spacing. The main producers are Spain (66% of EU production), Italy (15%), Greece (13%) and Portugal (5%). Spain has a leading position in the world in the production of olive oil (43% of the global production) [2]. One of the needed tasks for the agricultural business is the automation of the assessment of the size and health condition of olive trees (*Olea europaea* L.) for further forecast of the yield and profit. In addition, there are emerging threats that should be urgently addressed: the spread of the infection with the bacterium *Xylella fastidiosa Wells* et al. (1987) [3], and the effects of climate change such as the increase in extreme events (e.g., droughts, floods, and cold waves). These impacts affect photosynthesis, evapotranspiration, plant nutrition, and eventually plant growth and production [4,5]. Performing automatic monitoring of olive tree growth would be essential in these regions to effectively address these threats. Nowadays, the application of machine learning methods on very high spatial resolution satellite and aerial images opens the possibility of detecting isolated shrubs and trees at regional scale [6,7,8,9,10].

In precision agriculture, the use of unmanned aerial vehicle (UAV) images with the near-infrared (NIR), red, green, and blue spectral bands has been successfully incorporated for monitoring plant growth and status [11,12]. Spectral indices such as the normalized difference vegetation index (NDVI) or green normalized difference vegetation index (GNDVI) can be used to determine the type of crop, its performance, and its ripening stage [13]. GNDVI index is more sensitive to variation in crop chlorophyll content than the NDVI index, and GNDVI also has a higher saturation threshold, so it can be used in crops with dense canopies or in more advanced development stages and to evaluate moisture content and nitrogen concentrations in plant leaves [14]. On the other hand, NDVI index is particularly suitable for estimating crop vigor during the initial development stages [13,15].

On the other hand, deep learning methods in general and convolutional neural networks (CNNs) in particular have demonstrated impressive results over classical methods in extracting spatial patterns from natural RGB-images. In fact, CNNs constitute the state-of-the-art in all the fundamental computer vision tasks, in image classification [16], object detection and instance segmentation [17,18,19,20]. A good approach to accurately estimate olive tree crowns is by using instance segmentation models such as mask region-based convolutional neural networks (Mask R-CNN) [21], one of the most accurate CNN-based segmentation methods.

The main limitation of deep learning CNNs is that they require a large training dataset to achieve good results. In practice, in real world applications, several optimizations are used to overcome this limitation, namely, transfer learning, fine tuning, data augmentation [22], and potentially data-fusion.

The objective of this article is to illustrate the potential of deep CNNs for estimating the biovolume of olive-tree plantations from the tree crowns and shadows identified in ultra-high resolution images (less than a 30 cm, [23]). We first trained CNNs to identify olive tree crown and shadow segments. Then, we approximated tree biovolumes from the tree crown surfaces and the tree heights inferred from the shadow lengths. Previous works on shrubs and trees mainly focused on detection of plant species or damaged stages in unmanned aerial vehicle (UAV) images [6,24]. As far as we know, this is the first work in exploring the instance segmentation task for plant species segmentation with the objective of estimating the biovolume of trees.

The main contributions of this paper can be listed as follows:

- We have built a new annotated multi-spectral orthoimages dataset for olive tree crown segmentation, called OTCS-dataset. OTCS-dataset is organized into four subsets of different spectral bands and vegetation indices (RGB, NDVI, and GNDVI), at two spatial resolutions (3 cm/pixel and 13 cm/pixel).
- We evaluated the instance segmentation Mask R-CNN model for the tasks of olive trees crown segmentation and shadows segmentation in UAV images. We present a model based on the fusion of RGB images and vegetation indices that improves segmentation over models without image fusion.
- We estimated the biovolume of olive trees based on the area of their crowns and their height inferred from their shadow length.
- Our results show that NDVI or GNDVI spectral indices information with 13 cm/pixel resolution are enough for accurately estimating the biovolume of olive trees.

The paper is organized as follows: Related works are given in Section 2. The materials and methods are presented in Section 3, where the study area is shown in Section 3.1, the UAV RGB and multispectral images are shown in Section 3.2, the OTCSS-dataset construction is shown in Section 3.3, and the Mask R-CNN is presented in Section 3.4, the experimental setup is shown in Section 3.5, the metrics for CNN performance evaluation is in Section 3.6, and the biovolume calculation from tree crown and tree shadow estimations are shown in Section 3.7.

The experimental results are shown in Section 4, where the tree crown and tree shadow segmentation with RGB and vegetation indices images are provided in Section 4.1, and the results of tree biovolume calculations are presented in Section 4.2, Finally, Discussion and conclusions are provided in Section 5.

## 2. Related Works

**Figure 1:**
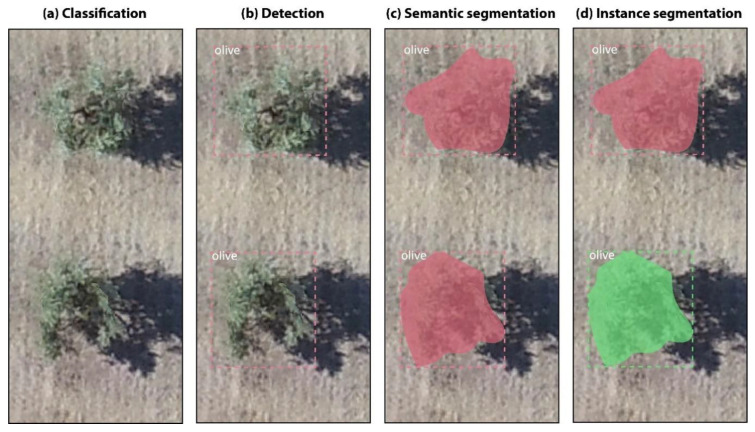
Illustration of the four fundamental computer vision tasks in the problem of olive-tree monitoring: (**a**) Image classification, (**b**) Object detection, (**c**) Semantic segmentation and (**d**) Instance segmentation.

Most problems in plant monitoring using high resolution remote sensing data are formulated as either: (a) an image classification problem, (b) an object detection problem, (c) a semantic segmentation problem or (d) an instance segmentation problem. In image classification, the method analyzes a given input image and outputs a label that describes the object-class existent in that image (see illustration in Figure 1a). In object detection, the method analyzes the input image and outputs a label together with a bounding box that delimits where the object-class is located in the image (Figure 1b). In semantic segmentation, the method analyzes the input image and outputs a label together with a polygon that delimits the pixels of each object-class (Figure 1c). In instance segmentation, the method analyzes the input image and outputs a label together with a polygon that delimits the pixels of each instance of the object-class (Figure 1d). Therefore, instance segmentation methods are potentially more suitable for estimating the surface of olive-tree crowns as they provide a precise estimation of all the pixels that constitute each olive-tree individual.

Unfortunately, the majority of the existing plant monitoring works reformulate their problems as either image classification tasks [24,25] or object detection tasks [26,27,28,29,30,31,32,33]. For example, the authors in [34] showed that applying a simple CNN-pixel-wise classification model on the fusion of high resolution digital surface model (DSM) with NDVI radiometric index provides a good potential for estimating crop/soil surface.

Few works address precision agriculture problems using deep learning segmentation methods. For example, for the estimation of pomegranate tree canopy in UAV images, the authors in [35] compared the performance of two CNN-based segmentation models, U-Net and Mask RCNN. Their experiments showed that faster RCNN achieved better results with respect to U-Net, with a mean average precision (mAP) of 57.5% versus 36.2%. In [8] the authors showed that the fusion of Mask-Fast RCNN and OBIA methods increases by 25% the overall accuracy of the segmentation of scattered shrubs in UAV, airborne and GoogleEarth imagery. In [36] the authors evaluated the performance of five CNN-based methods for the semantic segmentation of a single endangered tree species, called *Dipteryx alata* Vogel, in UAV images. In particular, they evaluated SegNet, U-Net, FC-DenseNet, and two DeepLabv3+ variants and found that FC-DensNet overcomes all the previous methods with an overall accuracy of 96.7%. In [37], the authors developed a CNN-based semantic segmentation method inspired by U-Net for the detection of mango tree individual crowns. Their experiment showed an overall accuracy of the order of 90%.

In the present paper, we will estimate olive tree biovolume from the tree crowns and tree shadows obtained by applying Mask R-CNN instance segmentation on ultra high resolution UAV images. Currently, Mask R-CNN is considered one of the most accurate deep CNN-based methods.

## 3. Materials and Methods

### 3.1. Study Area and UAV RGB and Multispectral Images

**Figure 2:**
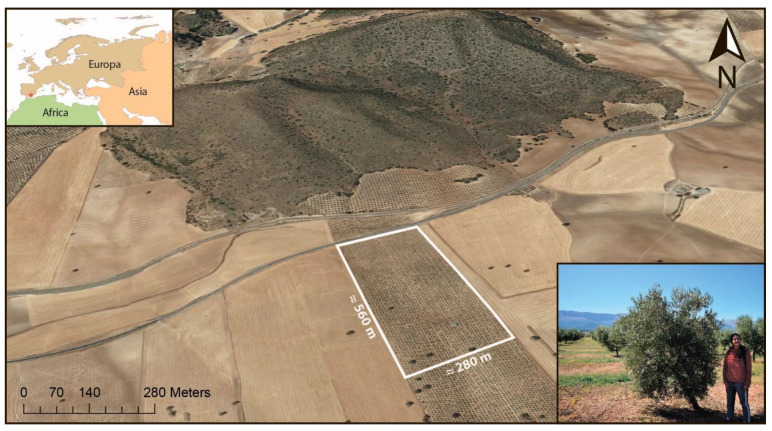
The test area in Andalusia, southern Spain (37°23′57″ N 3°24′47″ W).

The study area is located in Andalusia, Spain (37°23′57″ N 3°24′47″ W). The climate is Mediterranean, characterized by severe summer droughts and mild-wet winters. Average total annual precipitation is 400 mm and mean annual temperature is 15 ºC. This area is dominated by rainfed cereal croplands and olive groves in flatlands with some patches of natural vegetation in hills (Figure 2). To avoid competition for water availability among olive trees, they are separated by about 6 m from each other. The test area is within an olive grove of 50 hectares comprising 11,000 trees that were planted in 2006. We used a flat rectangle of 560 m × 280 m containing approximately 4000 trees as our study object.

### 3.2. UAV RGB and Multispectral Images

To compare the effect of deep learning models on different spatial and spectral resolutions, we made two UAV flights at 120 m height that captured an RGB image at ultra-high spatial resolution, and a multispectral image at very-high resolution:

(1) In February 2019, we flew a Sequoia multispectral sensor installed on the Parrot Disco-Pro AG UAV (Parrot SA, Paris, France) that captured four spectral bands (green, red, red edge, and near-infrared -NIR). The spatial resolution of the multispectral image was 13 cm/pixel. We then derived the vegetation indices detailed in the introduction: the normalized difference vegetation index (NDVI) (1) [38], and the green normalized difference vegetation index (GNDVI) (2) [14].
  (1) $$NDVI=\frac{NIR-Red}{NIR+Red},$$
  (2) $$GNDVI=\frac{NIR-Green}{NIR+Green}.$$
(2) In July 2019, to get finer spatial resolution, we flew the native RGB Hasselblad 20-megapixel camera of the DJI-Phantom 4 UAV (Parrot SA, Paris, France). The spatial resolution of the RGB image was 3 cm/pixel. These RGB images were then converted to 13-cm/pixel by spatial averaging so they could be compared to. In both flights, images were donated by the company Garnata Drone S.L. (Granada, Spain).

The specific conditions for the present data acquisition are weather conditions (sunny and cloudless day) and the time of shooting before sunset. For example, in our study, the following shots were made at 10:51, 9 February 2019 and at 18:54, 19 June 2019 (sunset on that day is at 20:27).

### 3.3. OTCSS-Dataset Construction

**Figure 3:**
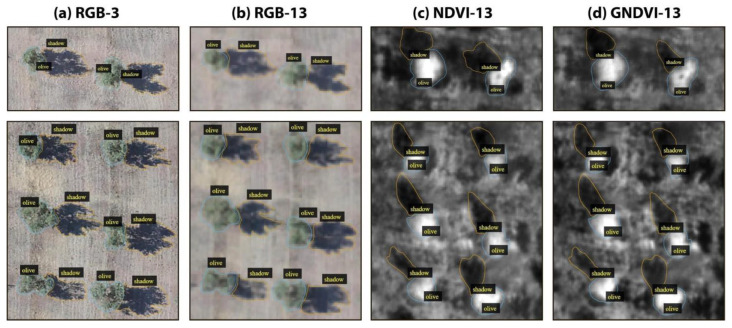
Examples of two image patches (first and second rows) in the four subsets of images (four columns) used to assess the effect of decreasing spatial resolution (RGB-3 versus RGB-13) and gaining spectral information (RGB-13 versus NDVI-13 OR GNDVI-13) for the task of instance segmentation of olive tree crowns and shadows in the OTCSS-dataset. (**a**) RGB-3 cm/pixel, (**b**) RGB-13 cm/pixel, (**c**) NDVI-13 cm/pixel and (**d**) GNDVI-13 cm/pixel. RGB: Red, Green, Blue; NDVI: normalized difference vegetation index; GNDVI: green normalized difference vegetation index.

To build a dataset for the task of instance segmentation of olive tree crowns and tree shadows that could let us assess the effect of decreasing spatial resolution and of gaining spectral information, we produced four subsets of data: (a) RGB-3, (b) RGB-13, (c) NDVI-13 and (d) GNDVI-13, where 3 and 13 indicate the spatial resolution of the images in cm/pixel (Figure 3). For each subset of data, we prepared 150 image patches that contained 2400 trees, of which 120 images (80% of the dataset) were used for training the model, and 30 images (20%) were used for testing the model on the olive tree crown class (Table 1) and on the olive tree shadow class (Table 2). Each image patch contained from one to eight olive trees with their corresponding tree crowns and tree shadows (see the example in Figure 3).

**Figure 4:**
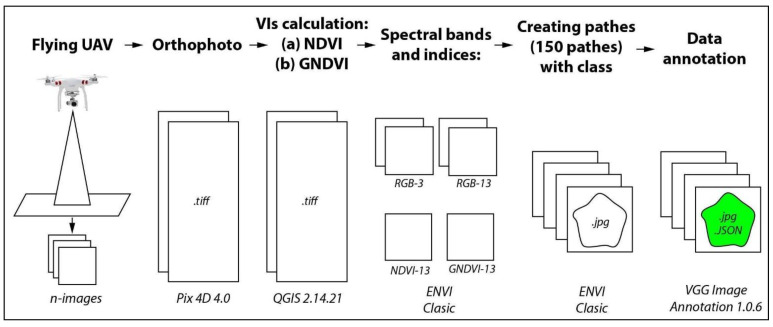
The process of preparing the images of OTCS-dataset.

The general scheme of creating the data set is shown in Figure 4. The original UAV images were mosaicked into an orthophoto by using Pix4D 4.0. QGIS 2.14.21 was used for reducing the spatial resolution of the RGB-3 cm/pixel to the resolution of RGB-13 cm/pixel, and for calculating the NDVI and GNDI indices. ENVI Classic was used for creating the patches and converting them from .tiff to .jpg format (the most suitable format for training deep learning models). During the .tiff to .jpg conversion the spatial resolution was artificially increased to 13 cm/pixel by QGIS 2.14.21 program. For creating and annotating the tree crown and the tree shadow segments in each image patch, we used VGG Image Annotator 1.0.6. which is a standalone software for manual annotation of images. The annotation process for this instance segmentation task was completely manual. That is, the annotator created a polygon surrounding each olive tree crown and another polygon surrounding each tree shadow instance. The created class labels with VGG annotator were then saved in a JSON format.

### 3.4. Mask R-CNN

The task of locating and delimiting all the pixels that constitute each individual olive tree crown in UAV images is called instance segmentation. This task is one of the most complex problems in computer vision. In this work we used the modern Mask R-CNN network (regions with convolutional neural networks) [8], which extends the faster R-CNN detection model [39]. Mask R-CNN analyzes an input image and provides three outputs for each object-class: (1) a class label that indicates the name of the object-class, (2) a bounding box that delimits each object-class and (3) a mask that delimits the pixels that constitute each object-class. For the considered problem in this work, Mask R-CNN generates for each olive tree a binary mask (with values 0 and 1), where value 1 indicates an olive tree pixel and 0 indicates a non-olive tree pixel.

Mask R-CNN is based on a classification model for the task of feature extraction. In this work, we used ResNet50 CNN [39] to extract increasingly higher-level features from the lowest to the deepest layer levels.

To further improve the generalization capacity of the segmentation model, we assessed the effect of the data augmentation technique [40], which consists of increasing the size of the dataset by applying simple transformations such as cropping (i.e., removing columns/rows of pixels at the sides of images), scaling, rotation, translation, horizontal and vertical shear. Instead of training Mask R-CNN (based on ResNet50) from scratch on our dataset, we used transfer learning, which consists of first initializing the weights of the model with pre-trained weights on a well known COCO-dataset [40], then retraining the model on our own dataset. The process of retraining the last years on a small new dataset is called fine tuning [22].

### 3.5. Experimental Setup

The preprocessing and training stages were carried out using Python programming language, version 3.5.2, and TensorFlow Object Detection API [41], an open-source software library for high-performance deep learning models. The calculations were performed on a computer with an Intel Xeon E5-2630v4 processor, accelerated using an NVIDIA Titan Xp graphics processor as a platform for learning and testing the proposed methodology. We used a learning rate of 0.001 and the stochastic gradient descent solver as an optimization algorithm. We trained Mask R-CNN network for 100 to 150 epochs on each different spectral bands and indices, i.e., RGB-3, RGB-13, NDVI-13, and GNDVI-13.

Thanks to transfer-learning from COCO and fine-tuning the execution time of the training process of Mask R-CNN on our dataset takes about half an hour on the GPU and several hours on the CPU. Testing Mask R-CNN over test images is very fast, almost real-time.

Several experiments were carried out to assess the effect of pixel size and the effect of using vegetation indices (that incorporate NIR information) instead of RGB images. We also quantified the benefit of using data augmentation on a small dataset. In total, we trained the following Mask R-CNN models:

- For tree crown estimation, we trained models on each subset of data separately (i.e., RGB-3, RGB-13, NDVI-13, and GNDVI-13) without (group A of models) and with data augmentation (group B of models) (i.e., scaling, rotation, translation, horizontal and vertical shear). In addition, we also tested whether data fusion could improve the generalization of the final model, that is, whether training a single model (model C) on all the RGB, NDVI, and GNDVI data together at 13 cm/pixel could result in a single general model able of accurately segmenting olive tree crowns independently of the input (i.e., RGB-13, NDVI-13, or GNDVI-13).
- For tree shadow estimation, we just trained one model (model D) with data augmentation on the RGB-3 subset to estimate tree heights on the dataset with highest spatial resolution precision. That model was then applied to the four subsets of data. In addition, we also tested whether data fusion could improve the generalization of the final model, that is, whether training a single model (model E) on all the RGB, NDVI, and GNDVI data together at 13 cm/pixel could result in a single general model able of accurately segmenting olive tree shadows independently of the input (i.e., RGB-13, NDVI-13, or GNDVI-13).

### 3.6. Metrics for CNN Performance Evaluation

To evaluate the performance of the trained Mask R-CNN on OCTS-dataset in the task of olive tree crown and shadow instance segmentation, we used the F1-score metric, which is defined as the harmonic mean of the precision and recall [42].

Mask R-CNN produces three outputs, a bounding-box, a mask, and a confidence about the predicted class. To determine whether a prediction is correct, the Intersection over union (*IoU*) or Jaccard coefficient [43] was used. It is defined as the intersection between the predicted bounding-box and actual bounding-box divided by their union. A prediction is true positive (*TP*) if *IoU* > 50%, and false positive (*FP*) if *IoU* < 50%. *IoU* is calculated as follows (3):

(3) $$IoU=\frac{Area\;of\;Overlap}{Area\;of\;Union}.$$

Usually, a threshold value of 0.5 is used, as it usually shows high indicators of scores [21]. The precision (4) and recall (5) are calculated as follows:

(4) $$Precision=\frac{TP}{TP+FP}=\frac{TP}{\#ground\_truths},$$

(5) $$Recall=\frac{TP}{TP+FN}=\frac{TP}{\#predictions}.$$

*Precision* determines the percentage of correctly recognized labels and *Recall* is part of a successful extraction of relevant labels.

F1-score is the weighted average of precision and recall (6). It takes both false positives and false negatives into account to ultimately measure the global accuracy of the model:

(6) $$F1=\frac{2\times Precision\times Recall}{Precision+Recall}.$$

### 3.7. Biovolume Calculation from Tree Crown and Tree Shadow Estimations

**Figure 5:**
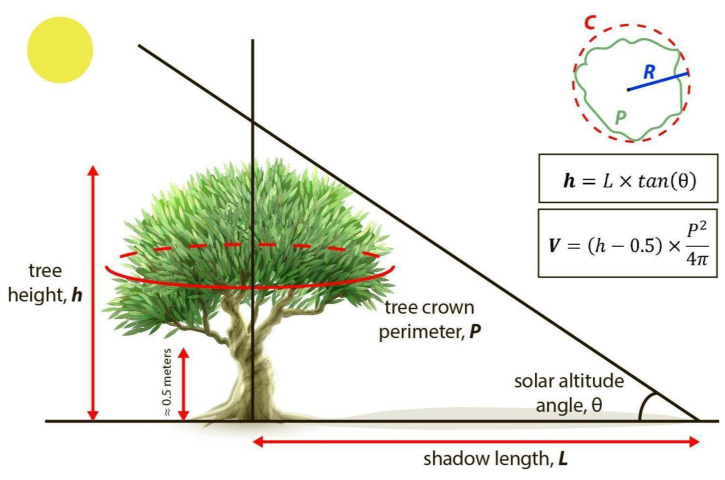
Illustration of the approximated biovolume in olive trees estimated from the automatic retrieval of tree crown and tree shadow polygons from deep neural networks (Mask R-CNN) applied on RGB or multispectral Unmanned Aerial Vehicle imagery. The volume of the tree canopy was approximated to a cylinder with a base of equal perimeter (*P* = *C*) to the polygon of the tree crown and with a height equal to the height (*h*) of the tree estimated from the length (*L*) of its shadow minus 0.5 m corresponding to the height of the unbranched trunk at the bottom. V: approximated biovolume; P: tree crown perimeter, equal to the circumference (*C*) of the cylinder base; *L*: length of tree shadow; *θ*: solar altitude angle; *h*: tree height. The olive tree picture was designed by macrovector and downloaded from www.freepik.com (accessed on 25 February 2021).

To estimate tree biovolumes from the tree crown and tree shadow polygons retrieved from the Mask R-CNN models outputs, we approximated it to a cylinder with a base of equal perimeter to the polygon of the tree crown and with a height equal to the height of the tree estimated from the length of its shadow minus 0.5 m corresponding to the height of the unbranched trunk:

- For tree crown surface (*S*), we first obtained the perimeter (*P*) of the tree crown polygon and then calculated the surface of a circle of the same perimeter.
- For tree height (*h*), we followed [44] to derive tree heights from tree shadows. In a flatland, the height of the tree (*h*) can be calculated from the length of the shadow (*L*) and the angle (*θ*) between the horizon and the sun altitude in the sky. The tree shadow length was derived from the shadow polygons as the distance from the tree crown polygon to the far end of the shadow polygon using QGIS 2.14.21 program. The angle between the horizon and the sun altitude can be calculated from the geographical position (latitude and longitude), date and time of imagery acquisition (7) [45]. Since the fly time and date of DL-Phantom 4 Pro drone was 10:51, 9 February 2019, and the coordinates were 37°23′57″ N 3°24′47″ W, the θ was 29.61°. The fly time and date of Parrot Disco-Pro AG was 18:54, 19 June 2019, and the coordinates were 37°23′57″ N 3°24′47″ W, the θ was 26.22° [46]:
  (7) $$h=L\times tan{\left(\theta \right)}_{;}$$
- Finally, for tree canopy volume (*V*), we approximated the biovolume in m^3^ by multiplying the tree crown surface (*S*) in m^2^ by the tree height minus 0.5 m (*L* − 0.5) in m. We systematically removed 0.5 m to the tree height to exclude the lower part of the tree trunk, on which there are no branches (on average about 0.5 m in height) (Figure 5). Though we could only take six ground truth samples for canopy biovolume, we assessed the overall accuracy of it as follows:
  (8) $$Accuracy=\left(1-\sum_{i=1}^{N}\frac{\left|V_{Gi}-V_{Mi}\right|}{V_{Gi}}\right)\times 100\%,$$
  where, *V_G_* is the approximate volume of tree canopy estimated from ground truth measurements, *V_M_* is the approximate volume of the tree canopy derived from the Mask R-CNN segmentation of tree crowns and shadows, *i* is each individual tree, and *N* is the total number of trees.

## 4. Experimental Results

This section has been divided into two parts. The segmentation results of the RGB and vegetation indices images are shown in Section 4.1. The results of tree biovolume calculations are presented in Section 4.2.

### 4.1. Tree Crown and Tree Shadow Segmentation with RGB and Vegetation Indices Images

**Figure 6:**
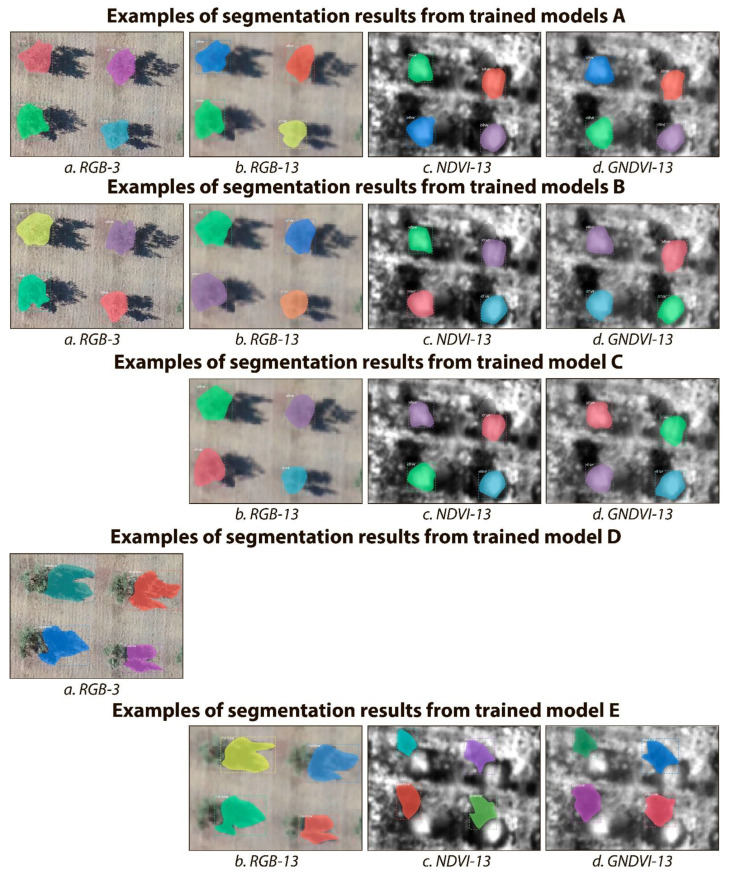
Examples of the segmentation results for the class “Olive tree crowns” (models A, B and C) and for the class “Olive tree shadows” (models D and E) using Mask R-CNN in the four image subsets of the OTCSS-dataset. See Section 3.4. *Experimental Setup* for model explanation. The testing datasets were: (**a**) RGB-3 cm/pixel, (**b**) RGB-13 cm/pixel, (**c**) NDVI-13 cm/pixel and (**d**) GNDVI-13 cm/pixel. RGB: Red, Green, Blue; NDVI: normalized difference vegetation index; GNDVI: green normalized difference vegetation index.

The performance, in terms of precision, recall, and F1-score, of all Mask R-CNN models on the corresponding test subsets of data are shown in Table 3 for tree crowns and in Table 4 for tree shadows. Graphical examples of the segmentation results of olive tree crowns and tree shadows are presented in Figure 6.

As shown in Table 3 for tree crown segmentation, all trained and tested Mask R-CNN models showed high *F1* score, above 94% across all subsets of data. Data augmentation did not significantly affect the *F1* score. The best performance (F1 = 100%) was reached with the RGB subset at a spatial resolution of 3 cm/pixel.

For the RGB datasets, coarsening the pixel size from 3 to 13 cm/pixel slightly decreased *F1* by 0.42% without data augmentation (models A) and by 0.86% with data augmentation (models B). At 13-cm/pixel resolution, the 3-band RGB images always produced greater *F1* scores than the single-band NDVI or GNDVI images. However, the model trained with data fusion (model C, which is trained on RGB, NDVI, and GNDVI images altogether) showed equivalent or greater *F1* than the models trained without data fusion (both with and without data augmentation, models A and B). For the NDVI-13 dataset, data fusion increased *F1* score by 1.76% while data augmentation decreased it by 2.68%, compared to training just with the NDVI-13 dataset and without data augmentation, respectively. The *F1* score reached on the GNDVI dataset was equivalent or greater than on the NDVI dataset.

As shown in Table 4 for tree shadow segmentation, all trained and tested Mask R-CNN models show a high *F1* score—above 96%. The highest *F1* score was reached for the model (model D) trained and tested on RGB images at 3 cm/pixel. However, the data fusion model (model E, which is trained on RGB, NDVI, and GNDVI images altogether) also showed very high *F1* on RGB-13 cm/pixel images (99.58%). The data fusion model (model E) performed better when tested on the RGB-13 (99.58%) and GNDVI-13 (98.73%) than on the NDVI-13 (96.10%) dataset for tree shadow segmentation.

### 4.2. Results of Tree Biovolume Calculations

Table 5 presents an example for the six olive trees that could be measured in the field for the approximation of free canopy volume from the tree perimeter and tree height segmentation obtained with the Mask R-CNN trained models. The overall accuracy was 94.51%, 75,61%, 82.58%, and 77,38% for RGB-3, RGB-13, NDVI-13, and GNDVI-13, respectively. The model trained and tested on RGB images at 3 cm/pixel showed the highest overall accuracy for biovolume estimation. At 13 cm/pixel scale, the data fusion model also performed well and reached better accuracy on the NDVI subsets than on the GNDVI or RGB subsets.

## 5. Discussion and Conclusions

The assessment of tree size with UAV imagery under the framework of precision agriculture could help the automatic monitoring of tree growing and performance, with large economic implications as in the case of olive production. Our results show how applying Mask R-CNN, both on RGB and vegetation indices imagery and both at 3 and 13 cm/pixel, can be used to accurately (*F1* always greater than 96%) map the crown and shadow segments of olive trees. These two polygons can then be used to estimate tree crown surface and tree height, two parameters commonly used to approximate tree canopy biovolume. Our test on six olive trees suggests that tree canopy biovolume can also be approximated (accuracy ranging from 77 to 95%) from these two CNN-derived parameters.

Currently, there are many affordable RGB and multispectral cameras that can be mounted on multi-rotor or fixed-wing drones and whose imagery can be automatically processed with CNN models for this purpose. On the one hand, RGB cameras mounted on a multi-rotor drone can capture much finer spatial resolution imagery, which increases accuracy of CNN models [8], but covering smaller areas (due to battery limitations), which results in more expensive imagery per hectare. On the other hand, multispectral cameras mounted on fixed-wing drones can capture coarser spatial resolution imagery but on larger areas, which decreases the cost per hectare, and with the benefit of incorporating plant reflectance in the near-infrared, and red-edge, which better relate to photosynthetic activity than just RGB [47]. Fusing both sources of data could join the advantage of both approaches, i.e., increase CNN accuracy, decrease the cost per hectare, and incorporate photosynthetic activity information [48]. Our results show that CNN models trained and tested at much finer resolution (i.e., RGB at 3 cm/pixel) reached slightly greater accuracy (only 0.42% more) than at coarser resolution (i.e., RGB at 13 cm/pixel). More importantly, our results show that training CNN models on the fusion of all RGB, NDVI and GNDVI subsets of images at coarser resolution (i.e., 13 cm/pixel resolution) enables to have a generalized model that maintains very high accuracies (always greater than 95% and 96% for tree crown and tree shadow, respectively) no matter the nature of the image (RGB, NDVI or GNDVI) used in the testing. This generalization opens the possibility of using fixed-wing multispectral or RGB imagery over extensive areas at a lower cost per hectare for the purpose of tree volume monitoring, with wide implications in precision agriculture, precision forestry and precision restoration.

Most sensors to obtain multispectral UAV imagery, such as the Parrot Sequoia used in this work, have four bands, i.e., green, red, red-edge, and near-infrared, but do not include a blue band to produce an RGB image [49]. Our results show that despite the absence of an RGB image, CNNs can reach very high accuracies just using the vegetation indices information (e.g., NDVI and GNDVI), if they are previously trained using a data fusion approach that incorporates both RGB and vegetation indices images. In other words, with a model trained in this way (RGB + NDVI + GNDVI) we could obtain greater precision in indices such as GNDVI which are usually obtained in flights with UAVs for precision agriculture. Furthermore, vegetation indices are widely used in agriculture around the world [13,50].

It is important to note that data augmentation when applying to the Mask R-CNN model did not affect much the results, and even tended to slightly decrease the F-1 score [51]. The best results among datasets with a resolution of 13 cm/pixel were achieved by models trained on the RGB image dataset, which may indicate that the model works best on three-band images, in contrast to single-band ones as with NDVI and GNDVI vegetation indices [52]. This can be explained by the fact that the augmentation data gave us some objects similar to the weeds that grow below and among the olive trees, which caused false positives and decreased the final F1. Despite this, our proof of concept shows how the method of pixel segmentation using deep CNNs can be used with high efficiency in problems of agriculture and forestry on UAV images.

Our illustration of how the CNN segmentation results of tree crown and tree shadow can be used to approximate biovolume in several trees is encouraging to investigate further in this sense to improve the method. The calculated values correspond well with the ground measurements of the test trees, showing minimum error of 5.4%. Additional field measurements, calculations, and experiments are needed to get a better understanding of the prospects of this approach, which is a task of further studies. In the future work, it is planned to conduct testing of trained CNN on satellite data of medium resolution, which is of the greatest interest for using possible results over large areas, as well as forecasting yields and profits from olive trees. Our approximation to estimate the biovolume can be very useful to automatically predict the yield and profit in terms of olive production especially if continuous monitoring of biovolume, given that yield per tree data is available. This method can also be extended to monitor tree foliage losses due to disturbances and annual canopy growth, which are useful to assess pruning treatments and for estimating production [53,54].

## Figures and Tables

**Table 1:** A brief description of the number of image patches and segments in the four subsets of the Olive Tree Crown Segmentation in the OTCSS-dataset: RGB-3 cm/pixel, RGB-13 cm/pixel, NDVI-13 cm/pixel, and GNDVI-13 cm/pixel. RGB: Red, Green, Blue; NDVI: normalized difference vegetation index; GNDVI: green normalized difference vegetation index.

Tree Crown Subset	# of Training Images	# of Training Segments	# of Testing Images	# of Testing Segments	Total of Images	Total of Segments
RGB-3	120	480	30	120	150	600
RGB-13	120	480	30	120	150	600
NDVI-13	120	480	30	120	150	600
GNDVI-13	120	480	30	120	150	600
Total	480	1920	120	480	600	2400

**Table 2:** A brief description of the number of image patches and segments in the four subsets of the Olive Tree Shadow Segmentation in the OTCSS-dataset: RGB-3 cm/pixel, RGB-13 cm/pixel, NDVI-13 cm/pixel, and GNDVI-13 cm/pixel. RGB: Red, Green, Blue; NDVI: normalized difference vegetation index; GNDVI: green normalized difference vegetation index.

Tree Shadow Subset	# of Training Images	# of Training Segments	# of Testing Images	# of Testing Segments	Total of Images	Total of Segments
RGB-3	120	480	30	120	150	600
RGB-13	120	480	30	120	150	600
NDVI-13	120	480	30	120	150	600
GNDVI-13	120	480	30	120	150	600
Total	480	1920	120	480	600	2400

**Table 3:** Segmentation performance of Mask R-CNN models for “Olive tree crown” class applied to the four subsets of the OTCSS-dataset in terms of *Precision*, *Recall* and F1-measure. *TP*: True Positive; *FP*: False Positive; FN: False Negative. The testing datasets were: RGB-3 cm/pixel, RGB-13 cm/pixel, NDVI-13 cm/pixel, and GNDVI-13 cm/pixel. RGB: Red, Green, Blue; NDVI: normalized difference vegetation index; GNDVI: green normalized difference vegetation index.

Testing Subset	*TP*	*FP*	*FN*	*Precision*	*Recall*	*F1*
*A.* *Trained models on each subset without data augmentation*						
RGB-3	120	0	0	1.0000	1.0000	**1.0000**
RGB-13	119	0	1	1.0000	0.9916	**0.9958**
NDVI-13	114	2	6	0.9827	0.9500	0.9660
GNDVI-13	110	0	10	1.0000	0.9166	**0.9564**
*B.* *Trained models on each subset with data augmentation*						
RGB-3	120	0	0	1.0000	1.0000	**1.0000**
RGB-13	118	0	2	1.0000	0.9833	0.9915
NDVI-13	118	13	2	0.9007	0.9833	0.9401
GNDVI-13	118	12	2	0.9076	0.9833	0.9439
*C.* *Trained models on the fusion of all 13-cm/pixel subsets of images and with data augmentation*						
RGB-13	119	0	1	1.0000	0.9916	**0.9958**
NDVI-13	116	0	4	1.0000	0.9666	**0.9830**
GNDVI-13	109	0	11	1.0000	0.9083	0.9519

**Table 4:** Segmentation performance of Mask R-CNN models for the “Olive tree shadow” class applied to the four subsets of the OTCSS-dataset in terms of *Precision*, *Recall* and F1-measure. *TP*: True Positive; *FP*: False Positive; FN: False Negative. The testing datasets were: RGB-3 cm/pixel, RGB-13 cm/pixel, NDVI-13 cm/pixel, and GNDVI-13 cm/pixel. RGB: Red, Green, Blue; NDVI: normalized difference vegetation index; GNDVI: green normalized difference vegetation index.

Testing Subset	*TP*	*FP*	*FN*	*Precision*	*Recall*	*F1*
*D.* *Trained models on each subset with data augmentation*						
RGB-3	120	0	0	1.0000	1.0000	**1.0000**
*E.* *Trained models on the fusion of all 13-cm/pixel subsets of images and with data augmentation*						
RGB-13	119	0	1	1.0000	0.9916	**0.9958**
NDVI-13	111	0	9	1.0000	0.9250	**0.9610**
GNDVI-13	117	0	3	1.0000	0.9750	**0.9873**

**Table 5:** The averaged characteristics by best trained models for 6 test olive trees, where *P* is the perimeter of the tree crown polygon used as the circumference of the cylinder base, *h* is the tree height derived from the tree shadow, *L* is the tree shadow length, *V* is the approximate volume of the tree canopy. *P*, *L*, and *h* are expressed in m; *V* is in m^3^. Models A (tree crown) and D (tree shadows) were trained and tested on RGB 3 cm/pixel images. Models C (tree crown) and E (tree shadow) were trained on a data fusion of the RGB, NDVI, and GNDVI altogether at 13 cm/pixel images but tested separately on each subset of data at 13 cm/pixel.

				Models A & D				Models C & E				Models C & E				Models C & E			
	Ground Truth			Tested on RGB-3				Tested on RGB-13				Tested on NDVI-13				Tested on GNDVI-13			
N	P	h	V	P	L	h	V	P	L	h	V	P	L	h	V	P	L	h	V
1	6.3	2.5	**6.31**	6.6	4.3	2.4	**6.70**	7.1	4.1	2.3	**7.34**	7.7	3.6	1.8	**6.00**	9.4	3.6	1.8	**8.95**
2	6.5	2.6	**7.06**	6.5	4.8	2.7	**7.40**	8.0	4.3	2.4	**9.89**	8.2	4.5	2.2	**9.18**	8.2	4.5	2.2	**9.18**
3	8.3	3.0	**13.70**	8.8	4.6	2.6	**13.02**	10.0	5.8	3.3	**22.25**	10.0	5.2	2.6	**16.4**	10.6	5.2	2.6	**18.42**
4	8.5	3.0	**14.37**	8.5	5.2	2.9	**14.11**	8.7	5.1	2.9	**14.34**	9.1	4.8	2.4	**12.28**	10.6	4.8	2.4	**16.66**
5	8.1	2.9	**12.53**	8.1	5.4	3.1	**13.41**	8.1	5.9	3.4	**14.89**	8.4	4.5	2.2	**9.63**	9.2	4.5	2.2	**11.56**
6	8.7	3.0	**15.05**	8.4	5.9	3.3	**16.02**	8.5	5.1	2.9	**13.78**	9.2	5.0	2.5	**13.21**	10.1	5.0	2.5	**15.93**

## Data Availability

All drone and airborne orthomosaic data, shapefile, and code will be made available on request to the corresponding author’s email with appropriate justification.

## Abbreviations

The following list of abbreviations was used in the manuscript:

CNN	Convolutional Neural Network
OTCSS	Olive Tree Crown and Shadow Segmentation dataset
UAV	Unmanned Aerial Vehicle
*IoU*	Intersection over Union
VGG	Visual Geometry Group
RGB	Red-Green-Blue
R-CNN	Regions-based CNN
NIR	Near-infrared
NDVI	Normalized Difference Vegetation Index
GNDVI	Green Normalized Difference Vegetation Index
GPU	Graphics Processing Unit
CPU	Central Processing Unit
